# Supplementary material for: Characteristics of a tattooed population and a possible role of tattoos as a risk factor for chronic diseases: Results from the LIFE-Adult-Study
Source: PLoS One. 2025 Sep 9;20(9):e0319229. doi: 10.1371/journal.pone.0319229 (PMC12419626; doi:10.1371/journal.pone.0319229)
Supplement: S1 Questionnaire — (PDF) [file pone.0319229.s001.pdf]

S1 Questionnaire. Tattoo-Questionnaire (original in German).

Fragebogen über das Vorhandensein einer Tätowierung oder eines Permanent Make-ups

1. Bitte kreuzen Sie Zutreffendes an und füllen die für Sie in Frage kommenden Felder aus:

	**Ja**	**Nein**	**Wenn ja, wann haben Sie die erste Tätowierung/Permanent Make-up erhalten?** *(Angabe in Monat und Jahr; z.B.: 04.2004 für April 2004)*
**Haben Sie eine Tätowierung?**	☐	☐	☐☐.☐☐☐☐ **Monat** **Jahr**
**Haben Sie Permanent Make-up?**	☐	☐	☐☐.☐☐☐☐ **Monat** **Jahr**

Falls Sie ***keine*** *Tätowierung(en) und/oder Permanent Make-up haben, endet der Fragebogen hier für Sie.*

Beantworten Sie die folgenden Fragen bitte, wenn Sie Tätowierungen und/oder Permanent Make-up haben. Kreuzen Sie bitte die für Sie zutreffenden Antworten an.

2. Bitte machen Sie Angaben zur Farbe Ihrer Tätowierungen/Ihres Permanent Make-ups.

	**Weiß**	**Gelb**	**Orange**	**Rot**	**Magenta**	**Violett**	**Blau**	**Gün**	**Braun**	**Schwarz**
**Welche Farbe hat Ihre Tätowierung / haben Ihre Tätowierungen?**	☐	☐	☐	☐	☐	☐	☐	☐	☐	☐
**Welche Farbe hat Ihr Permanent Make-up?**	☐	☐	☐	☐	☐	☐	☐	☐	☐	☐

3. Bitte geben Sie anhand der Auswahlmöglichkeiten in der Tabelle Auskunft über Lokalisation und ungefähre Größe Ihrer Tattoos oder Ihres Permanent Make-ups.

**Körperteil**	**Größe des Tattoos /des Permanent Make-up (ungefährer Durchmesser)**			
	kleiner als 5 cm	5 bis 10 cm	10 bis 20 cm	größer als 20 cm
**Gesicht**	☐	☐	☐	☐
**anderer Teil des Kopfes (außer Gesicht)**	☐	☐	☐	☐
**Hals**	☐	☐	☐	☐
**Rumpf, Vorderseite - Bereich oberhalb der Taille**	☐	☐	☐	☐
**Rumpf, Vorderseite Bereich unterhalb der Taille**	☐	☐	☐	☐

**Körperteil**	**Größe des Tattoos /des Permanent Make-up (ungefährer Durchmesser)**			
	kleiner als 5 cm	5 bis 10 cm	10 bis 20 cm	größer als 20 cm
**Rücken, oberhalb der Taille**	☐	☐	☐	☐
**Rücken, unterhalb der Taille**	☐	☐	☐	☐
**rechter Oberarm**	☐	☐	☐	☐
**linker Oberarm**	☐	☐	☐	☐
**rechter Unterarm/Handgelenk**	☐	☐	☐	☐
**linker Unterarm/Handgelenk**	☐	☐	☐	☐
**rechte Hand**	☐	☐	☐	☐
**linke Hand**	☐	☐	☐	☐
**rechter Oberschenkel, Vorderseite**	☐	☐	☐	☐
**linker Oberschenkel, Vorderseite**	☐	☐	☐	☐
**rechter Oberschenkel, Rückseite**	☐	☐	☐	☐
**linker Oberschenkel, Rückseite**	☐	☐	☐	☐
**rechter Unterschenkel/Fußknöchel, Vorderseite**	☐	☐	☐	☐
**linker Unterschenkel/Fußknöchel, Vorderseite**	☐	☐	☐	☐
**rechter Unterschenkel, Rückseite**	☐	☐	☐	☐
**linker Unterschenkel, Rückseite**	☐	☐	☐	☐
**rechter Fuß**	☐	☐	☐	☐
**linker Fuß**	☐	☐	☐	☐
**andere Körperregion, Welche? ____________________**	☐	☐	☐	☐

4. Haben Sie sich jemals einer Laserbehandlung unterzogen, um eine Tätowierung entfernen zu lassen?

☐ nein ☐ ja Wenn ja, wann? ☐☐.☐☐☐☐

Monat **Jahr**

5. Haben oder hatten Sie irgendwelche medizinischen Beschwerden bezüglich Ihrer Tätowierung oder Ihres Permanent Make-ups?

☐ nein ☐ ja Wenn ja, welche?

☐ Allergische Hautreaktionen

☐ Juckreiz/Schmerz/Schwellung

☐ Hautinfektionen

☐ Wucherungen /Tumore

☐ Andere: ________________

6. Treten bei Ihnen Hautreizungen auf, wenn Sie mit Jeans-Knöpfen, Armbändern, Ringen, Schmuck oder ähnlichen Gegenständen in Berührung kommen? Wenn ja, seit wann?

☐ nein ☐ ja Wenn ja, seit wann? ☐☐.☐☐☐☐

Monat **Jahr**

8. Haben Sie nach Erhalt Ihrer ersten Tätowierung Sonnenstudios oder Solarien besucht?

☐ nein ☐ ja
