# Supplementary figures and images for: Characteristics of a tattooed population and a possible role of tattoos as a risk factor for chronic diseases: Results from the LIFE-Adult-Study
Source: PLoS One. 2025 Sep 9;20(9):e0319229. doi: 10.1371/journal.pone.0319229 (PMC12419626; doi:10.1371/journal.pone.0319229)

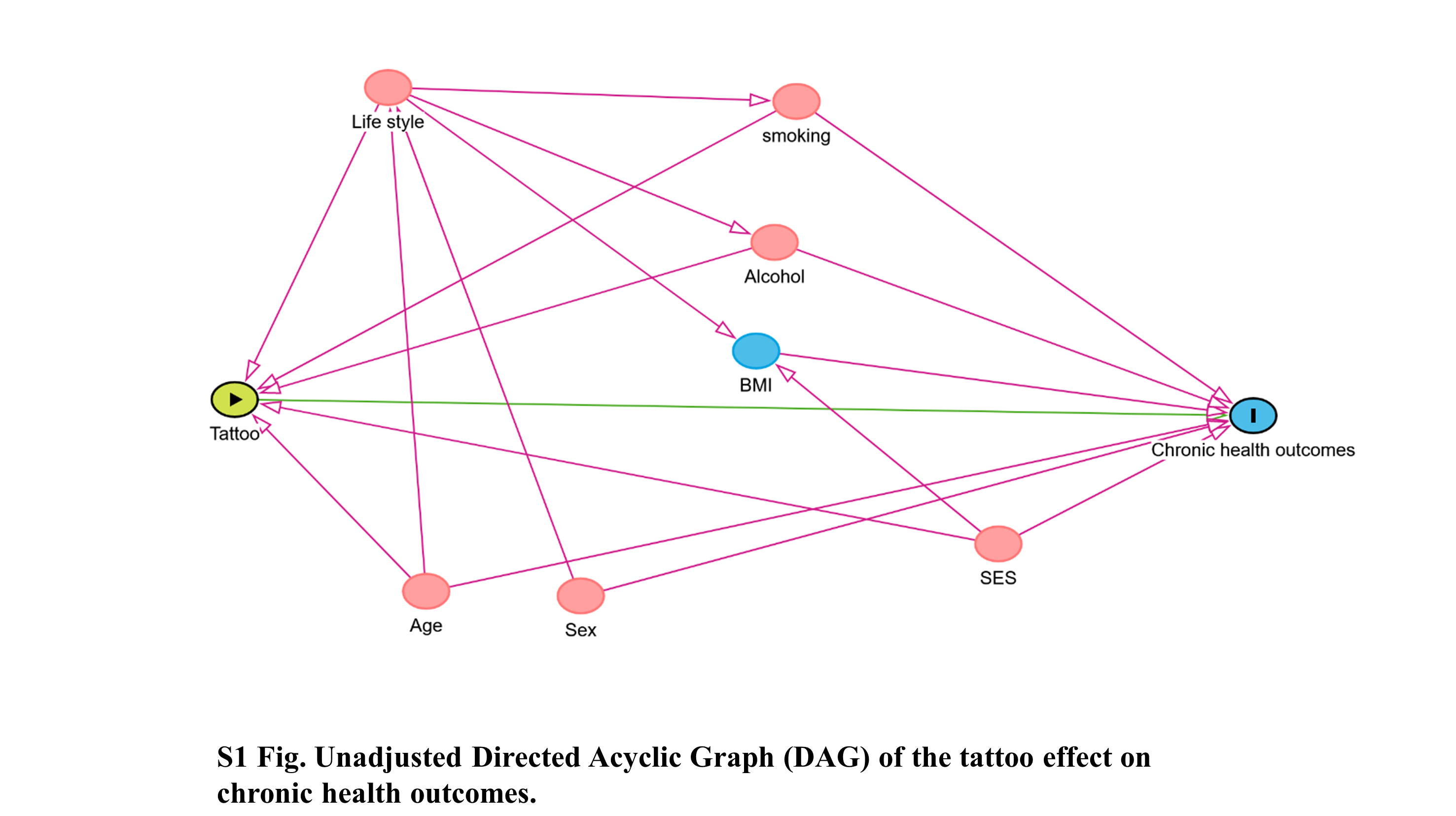

Supplement: S1 Fig — (TIF) [file pone.0319229.s002.tif]

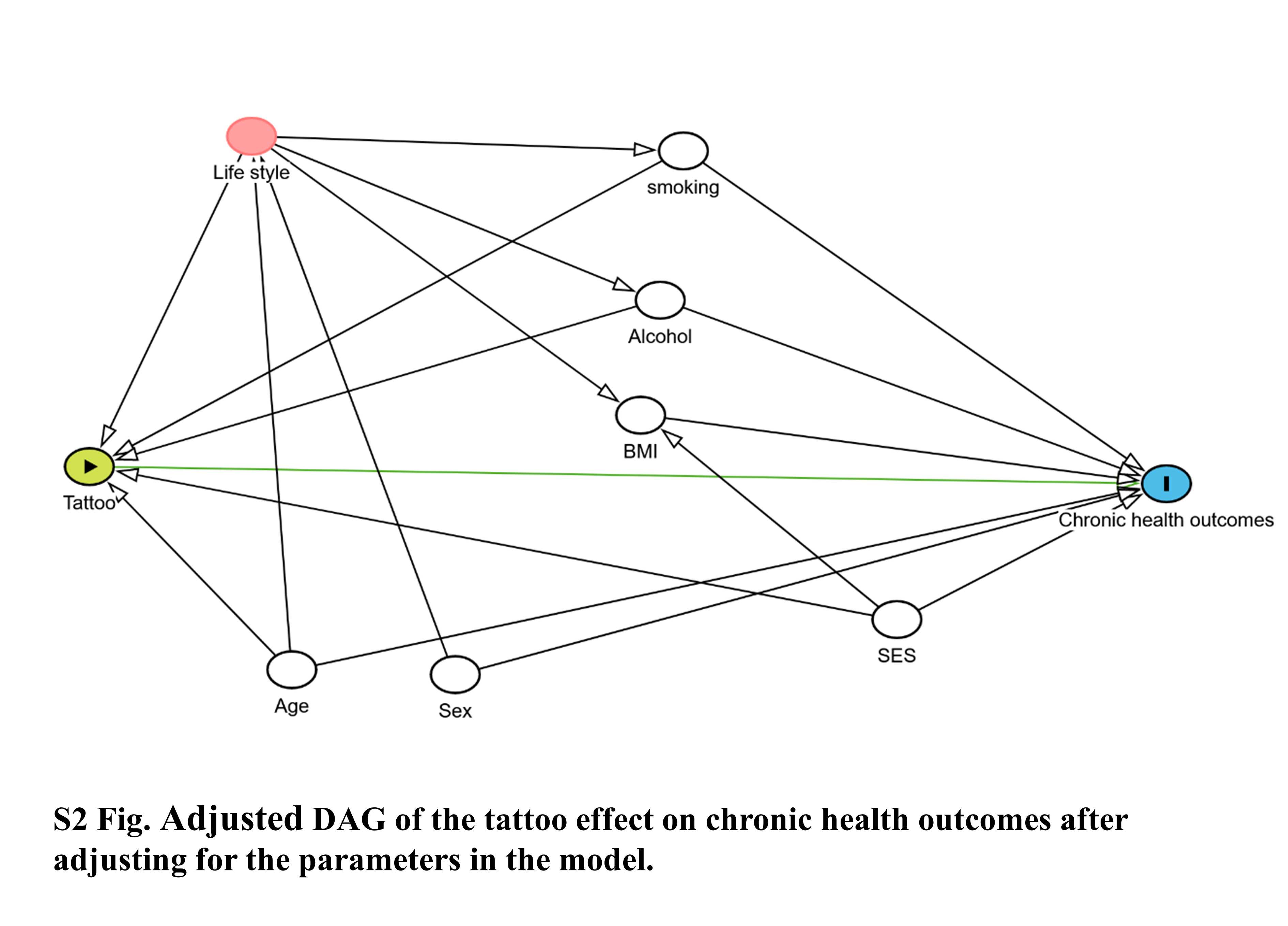

Supplement: S2 Fig — (TIF) [file pone.0319229.s003.tif]

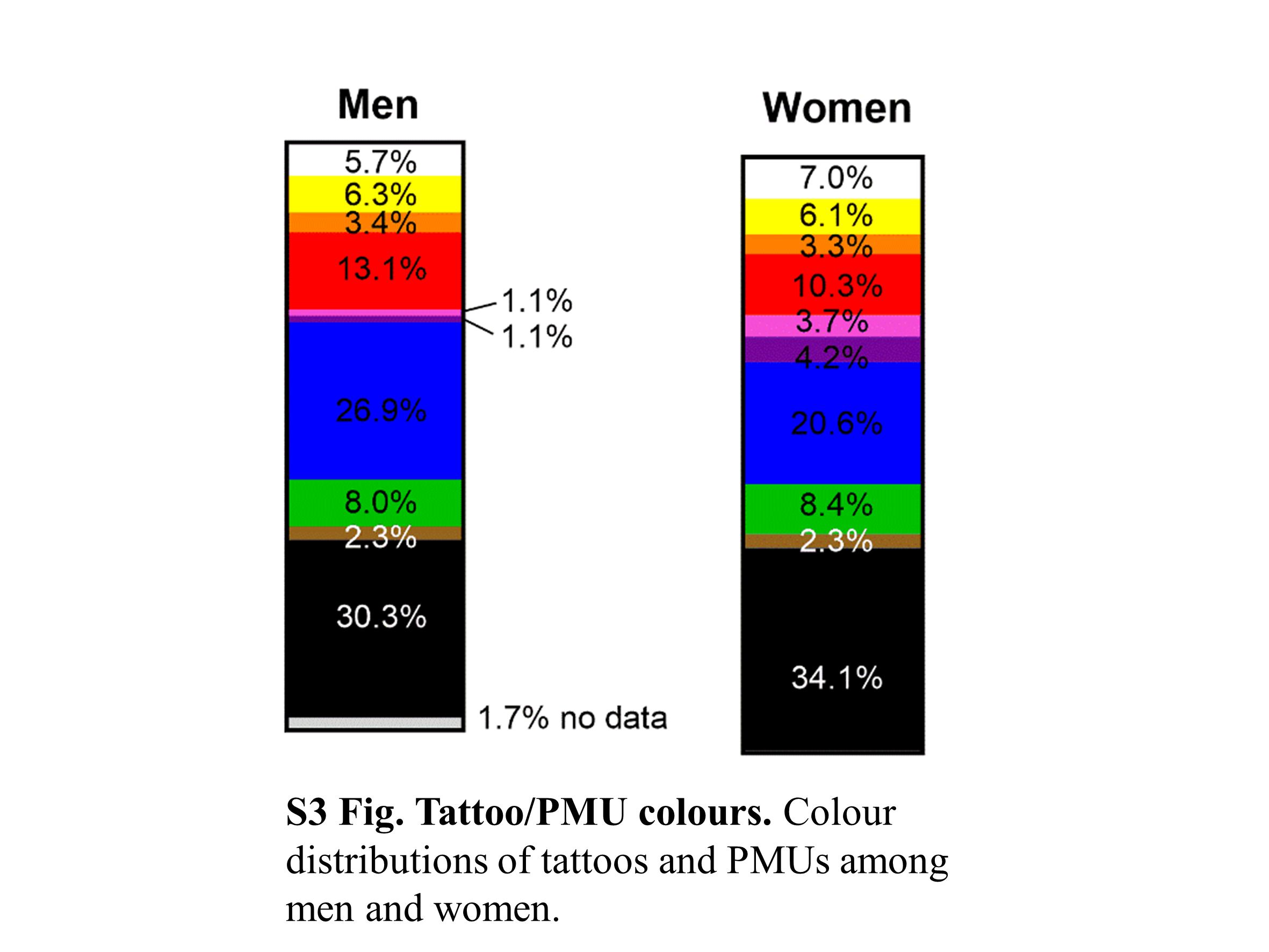

Supplement: S3 Fig — Colour distributions of tattoos and PMUs among men and women. (TIF) [file pone.0319229.s004.tif]

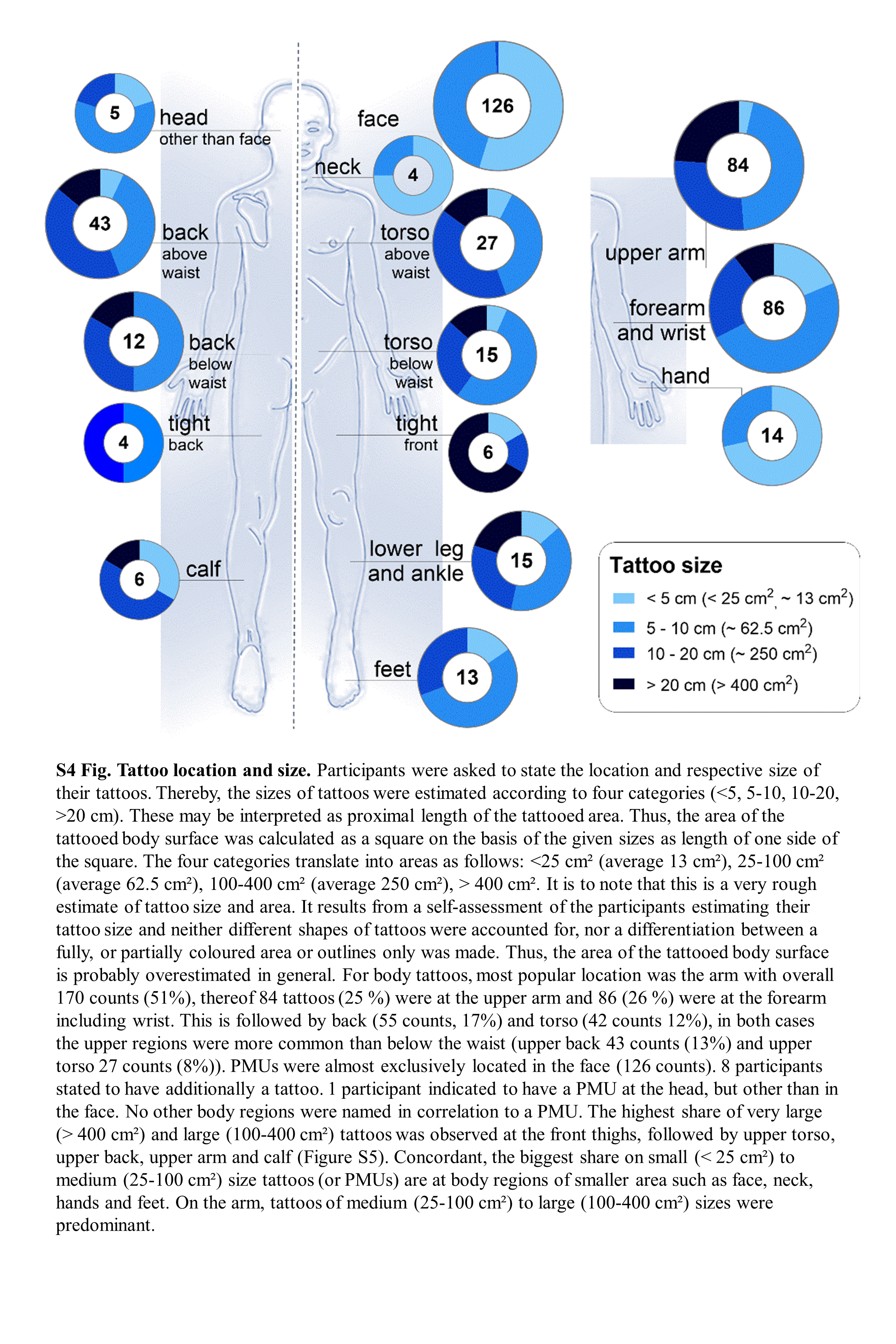

Supplement: S4 Fig — Participants were asked to state the location and respective size of their tattoos. Thereby, the sizes of tattoos were estimated according to four categories (<5, 5–10, 10–20, > 20 cm). These may be interpreted as proximal length of the tattooed area. Thus, the area of the tattooed body surface was calculated as a square on the basis of the given sizes as length of one side of the square. The four categories translate into areas as follows: < 25 cm² (average 13 cm²), 25–100 cm² (average 62.5 cm²), 100–400 cm² (average 250 cm²), > 400 cm². It is to note that this is a very rough estimate of tattoo size and area. It results from a self-assessment of the participants estimating their tattoo size and neither different shapes of tattoos were accounted for, nor a differentiation between a fully, or partially coloured area or outlines only was made. Thus, the area of the tattooed body surface is probably overestimated in general. For body tattoos, most popular location was the arm with overall 170 counts (51%), thereof 84 tattoos (25%) were at the upper arm and 86 (26%) were at the forearm including wrist. This is followed by back (55 counts, 17%) and torso (42 counts 12%), in both cases the upper regions were more common than below the waist (upper back 43 counts (13%) and upper torso 27 counts (8%)). PMUs were almost exclusively located in the face (126 counts). 8 participants stated to have additionally a tattoo. 1 participant indicated to have a PMU at the head, but other than in the face. No other body regions were named in correlation to a PMU. The highest share of very large (> 400 cm²) and large (100–400 cm²) tattoos was observed at the front thighs, followed by upper torso, upper back, upper arm and calf (S5 Fig). Concordant, the biggest share on small (< 25 cm²) to medium (25–100 cm²) size tattoos (or PMUs) are at body regions of smaller area such as face, neck, hands and feet. On the arm, tattoos of medium (25–100 cm²) to large (100–400 cm²) sizes were predomina [file pone.0319229.s005.tif]

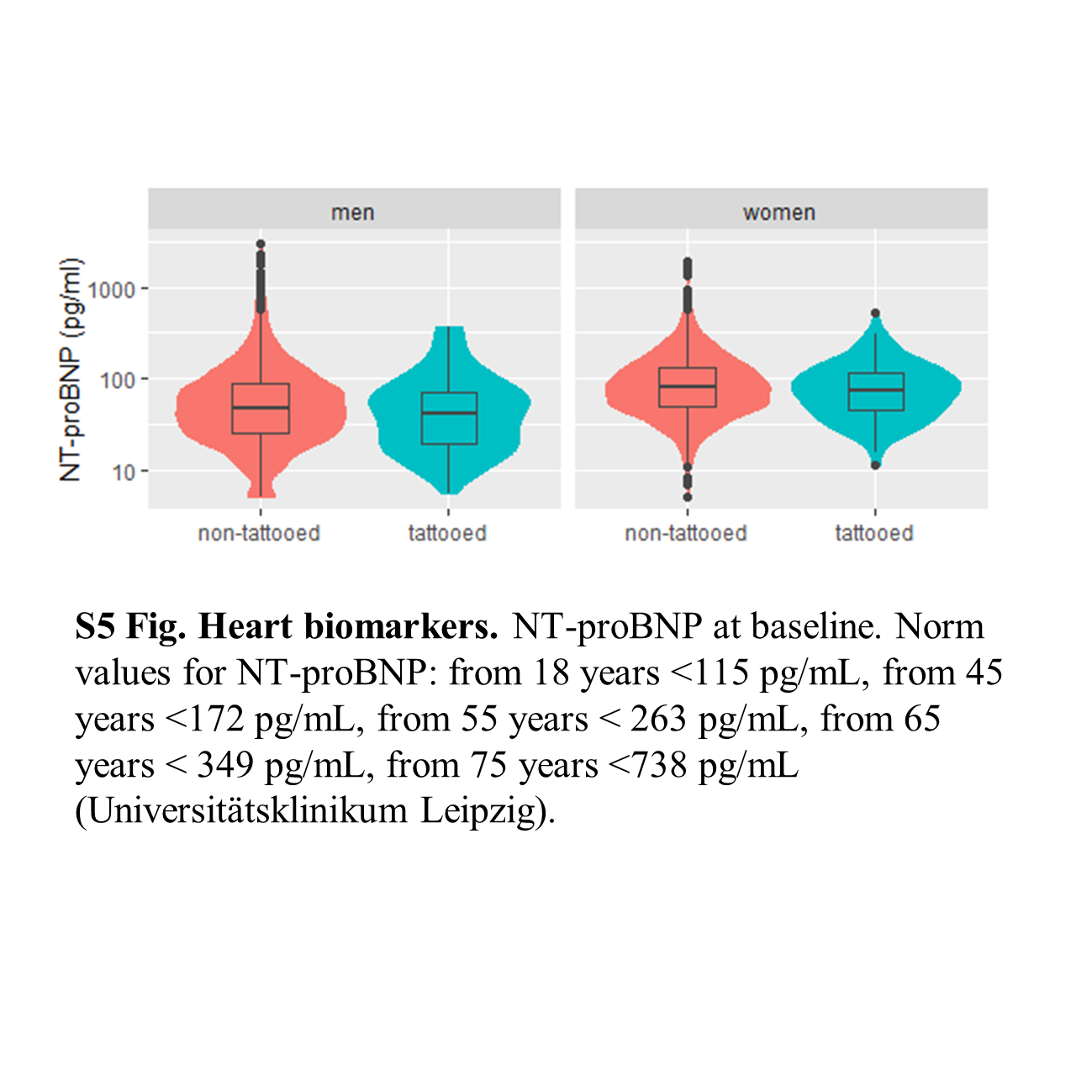

Supplement: S5 Fig — NT-proBNP at baseline. Norm values for NT-proBNP: from 18 years <115 pg/mL, from 45 years <172 pg/mL, from 55 years < 263 pg/mL, from 65 years < 349 pg/mL, from 75 years <738 pg/mL (Universitätsklinikum Leipzig). (TIF) [file pone.0319229.s008.tif]

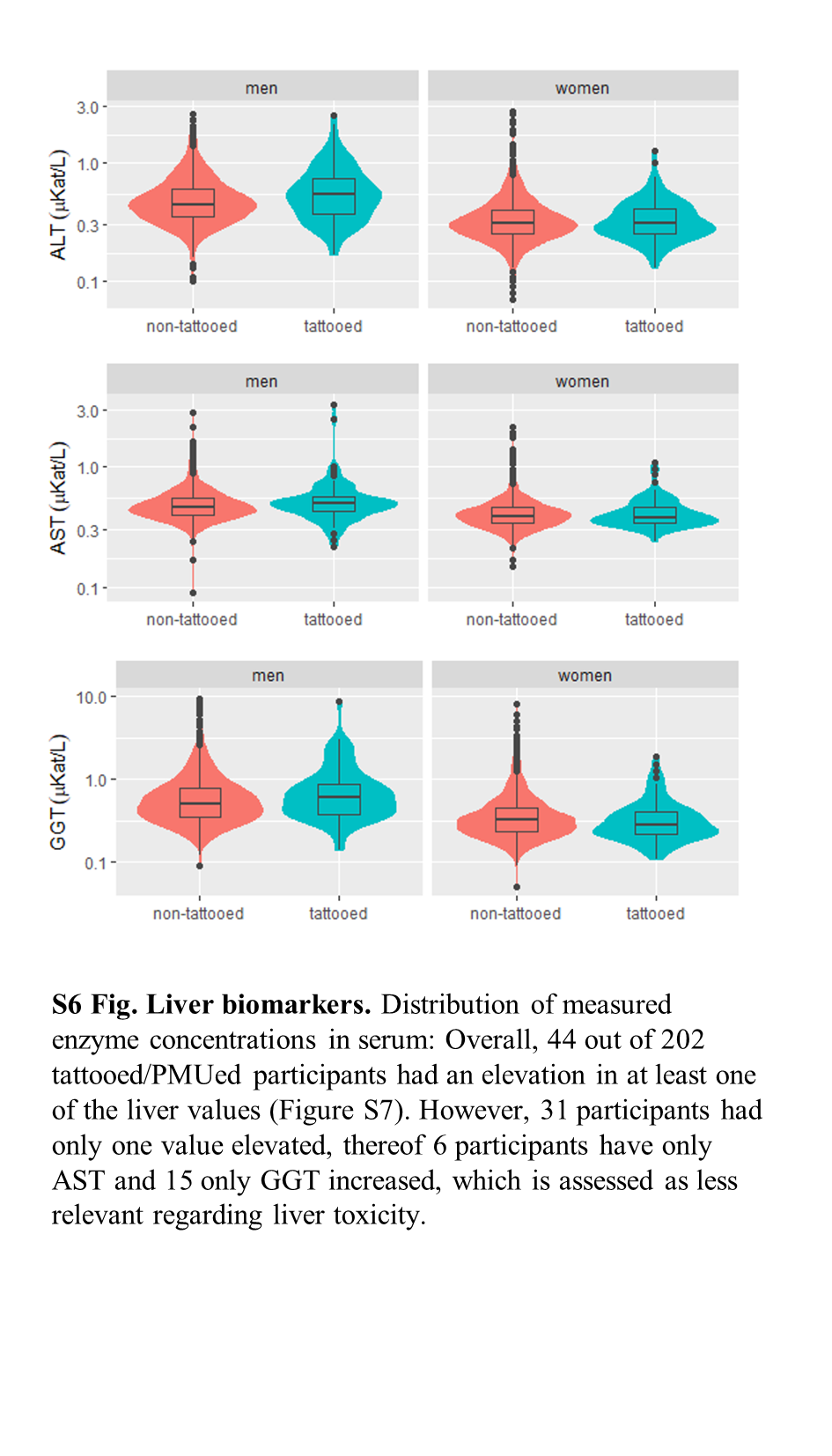

Supplement: S6 Fig — Distribution of measured enzyme concentrations in serum: Overall, 44 out of 202 tattooed participants had an elevation in at least one of the liver values (Figure S7). However, 31 participants had only one value elevated, thereof 6 participants have only AST and 15 only GGT increased, which is assessed as less relevant regarding liver toxicity. (TIF) [file pone.0319229.s009.tif]

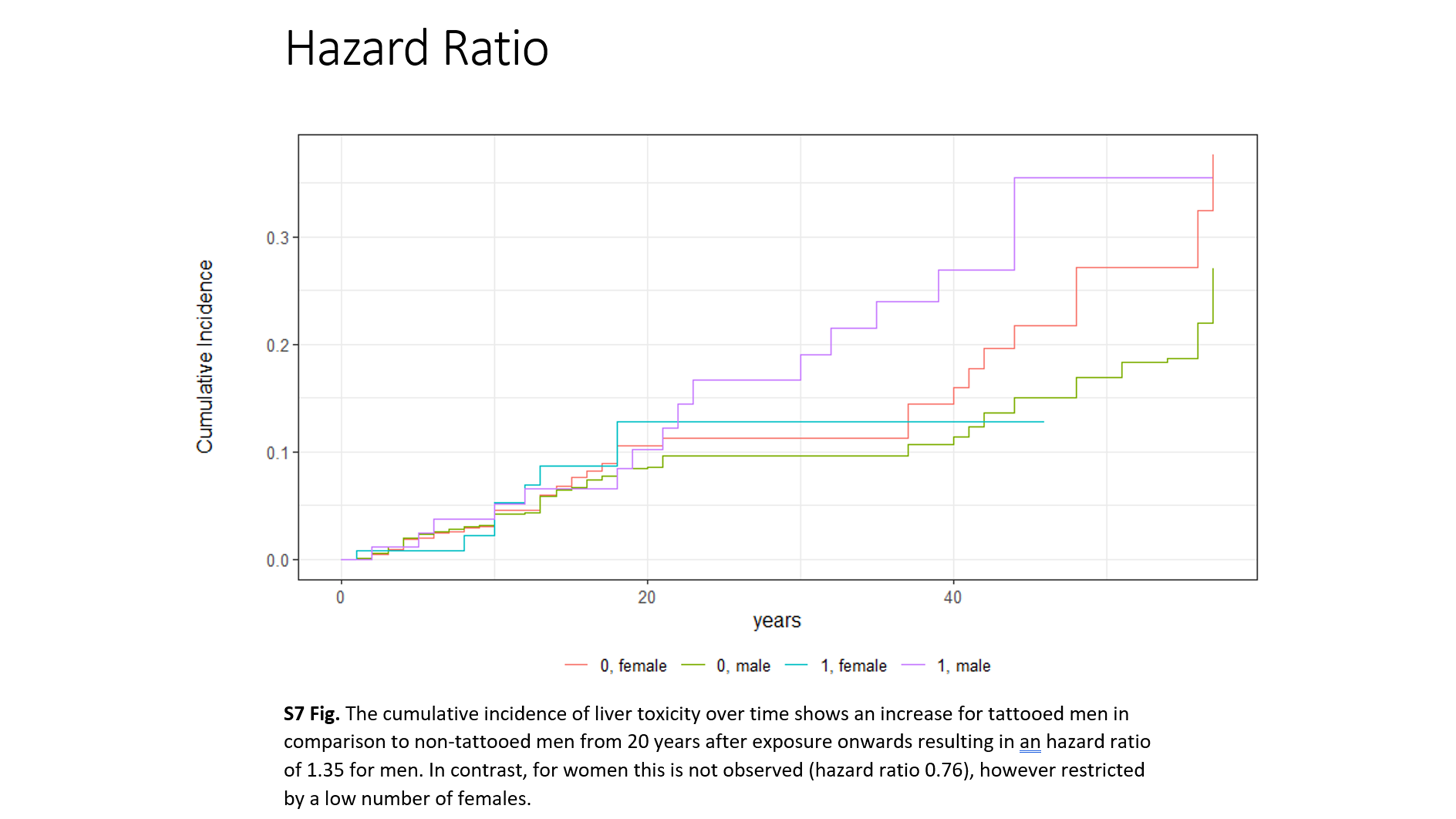

Supplement: S7 Fig — (TIF) [file pone.0319229.s010.tif]

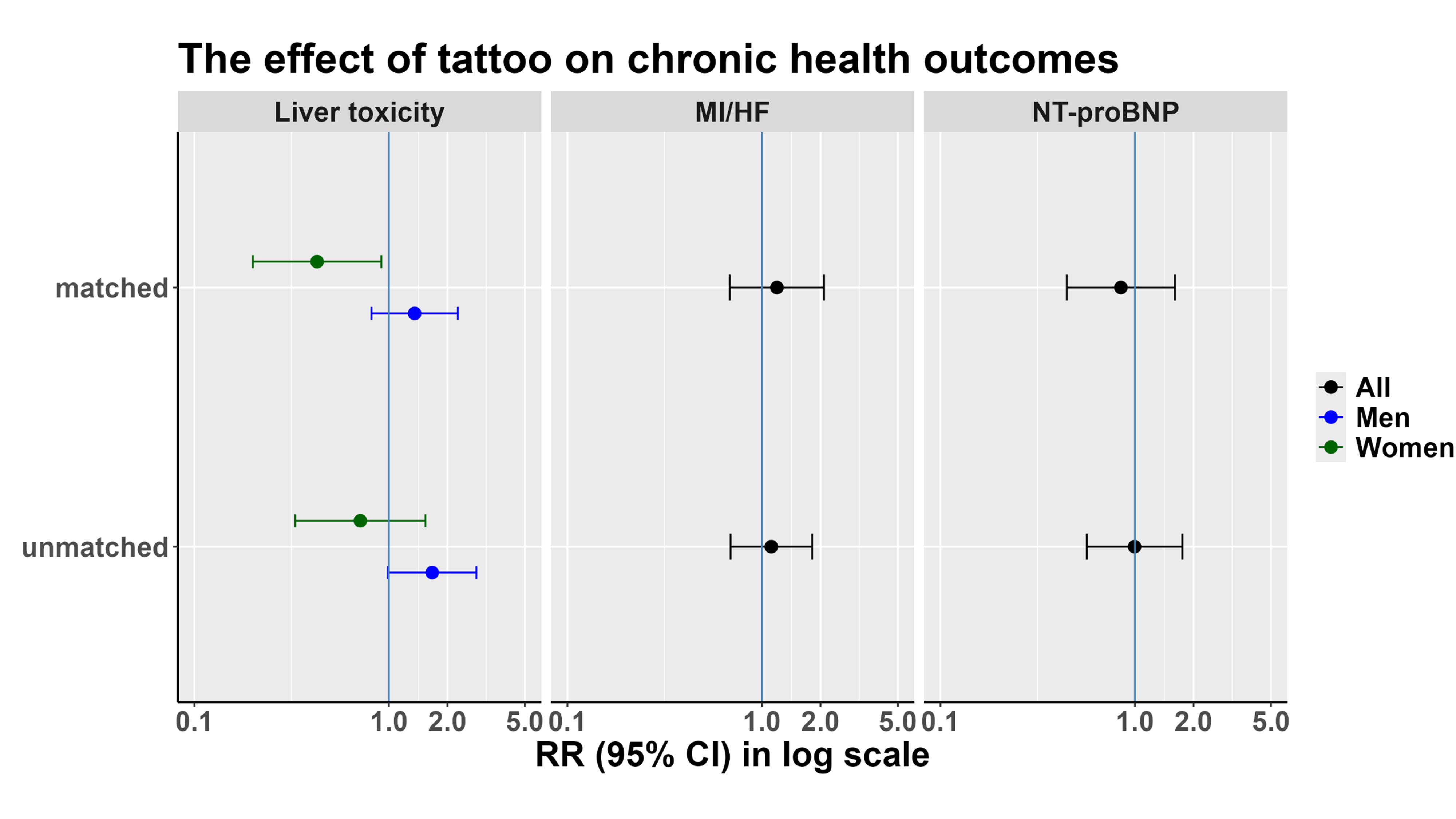

Supplement: S8 Fig — (TIF) [file pone.0319229.s011.tif]
