# Supplementary material for: Characteristics of a tattooed population and a possible role of tattoos as a risk factor for chronic diseases: Results from the LIFE-Adult-Study
Source: PLoS One. 2025 Sep 9;20(9):e0319229. doi: 10.1371/journal.pone.0319229 (PMC12419626; doi:10.1371/journal.pone.0319229)
Supplement: S1 Table — (PDF) [file pone.0319229.s006.pdf]

S1 Table. Medical issues related to tattoos and PMUs (self-estimation of participants).

Medical issue related to tattoo/PMU (n=16)														
**pain, itchiness or swelling**	**infection**	**allergic reaction**	**tumors**	**other**	**Tattoo or PMU (year)**	**Tattooing extent**	**Colors of T/PMU**	**Allergy against nickel indicated? (tattoo quest.)**	**solariums visited?**	**Men (M)/ Women (W)**	**Age in years**	**Smoking? [1]**	**BMI Cat.[1]**	**Diseases occurred after T/PMU according to medical Anamnese[1,2]**
10/16					PMU: 8/10 (2010-2018)<br>Tattoo:2/10 (2002-2006)	PMU: Small<br>Tattoo: small and very large	PMU: bl (5/8) br (4/8)<br>Tattoo: bl (2/2) y (2/2), m (2/2), w (1/2), r (1/2), v (1/2), b (1/2)	1/10 (prior to T/PMU)	3/10	M: 1/10<br>W: 9/10	median (IQR): 70 (18)	n: 6/10<br>f: 2/10<br>c: 1/10<br>n/a: 1/10	Median (IQR): 2 (1)	thyroid hypofunction, migraine, elevated blood lipids; Asthma, kidney stone, thyroid struma and Hashimoto, glaucoma
	3/16 (PMU)				PMU: 3/3 (2011-2018)<br>Tattoo: 2/3 (1996-2007)	PMU: Small-large	PMU: m (1/3), r (2/3), br (2/3), bl (2/3)<br>Tattoo: v (2/2), bl (2/2), w (1/2), m (1/2), v (1/2), b (1/2), g (1/2)	1/3 (prior to T/PMU	1/3	M: 0/3<br>W: 3/3	62 (range: 52-66)	f: 3/3	Median (IQR): 3 (1)	-
		2/16			PMU: 1/2 (2017)<br>Tattoo: 1/2 (2006)	PMU: small<br>Tattoo: very large	PMU: br (changed to r)<br>Tattoo: w, y, o, r, b, g, br, bl	0/2	1/2	M :1/2<br>W: 1/2	37 and 67 years	n: 2/2	2 and 3	thyroid hypofunction and migraine
			0/16											
				2/16	Tattoo 2/2 (2005 -2016)	Medium 2/2	bl (2/2), w (1/2), o (1/2), r (1/2), b (1/2)	1/2 (prior to T)	1/2	M: 2/2<br>W: 0/2	60 and 68	n: 1/2	2	thyroid – (Hashimoto)

Colors: white (**w**), yellow (**y**), orange (**o**), red (**r**), magenta (**m**), violet (**v**), blue (**b**), green (**g**), brown (**br**), black (**bl**); **Smoking**: n – non-smoker, f- former smoker –c current smoker; [1] – data from basic examination; [2]-data from follow-up questionnaires; n/a – no answer
