# Supplementary material for: Characteristics of a tattooed population and a possible role of tattoos as a risk factor for chronic diseases: Results from the LIFE-Adult-Study
Source: PLoS One. 2025 Sep 9;20(9):e0319229. doi: 10.1371/journal.pone.0319229 (PMC12419626; doi:10.1371/journal.pone.0319229)
Supplement: S2 Table — (PDF) [file pone.0319229.s007.pdf]

S2 Table. Characteristics of tattooed participants in comparison to non-tattooed/ for analysis of cardiologic diseases and prevalence of MI and HF.

**Characteristic**	**Overall**, N = 4,249[1]	**no-tattoo**, N = 3,972[1]	**tattooed**, N = 278[1]
**Prevalence MI**	127/4249 (3.0%)	120/3972 (3.0%)	7/278 (2.5%)
**Prevalence HF**	174/4249 (4.1%)	163/3972 (4.1%)	11/278 (4.0%)
**Prevalence combined MI/HF**		265/3972 (6.7%)	15/278 (5.4%)
**Tattooing extent**			Small- 7/15<br>Medium – 5/15<br>Large – 1/15
**Median age at first cardiologic event**		59.5 years (IQR: 15 years)	61 years (IQR: 14 years)
**Gender**			
Men	2,044 (48%)	1,954 (49%)	90 (32%)
Women	2,205 (52%)	2,018 (51%)	187 (68%)
**Age**	69 (58, 78)	70 (59, 78)	61 (54, 72)
**Age categorized**			
Elderly (> 60)	2,908 (68%)	2,768 (70%)	140 (51%)
Adult (40-60)	1,230 (29%)	1,108 (28%)	122 (44%)
Young (20-40)	111 (2.6%)	96 (2.4%)	15 (5.4%)
**Body Mass Index (BMI)**	26.4 (23.9, 29.7)	26.5 (24.0, 29.7)	25.0 (22.9, 28.8)
BMI categorized			
Underweight	17 (0.4%)	15 (0.4%)	2 (0.7%)
Normal	1,508 (35%)	1,373 (35%)	135 (49%)
Overweight	1,749 (41%)	1,659 (42%)	90 (32%)
Obese	970 (23%)	920 (23%)	50 (18%)
Unknown	5 (0.1%)	5 (0.1%)	0 (0%)
**Socioeconomic status (SES)**			
Low	621 (15%)	573 (14%)	48 (17%)
Medium	2,585 (61%)	2,405 (61%)	180 (65%)
High	1,032 (24%)	984 (25%)	48 (17%)
Unknown	11 (0.3%)	10 (0.3%)	1 (0.4%)
**Alcohol consumption (g/day)**	5 (1, 16)	5 (1, 16)	4 (1, 14)
**Alcohol consumption (categorized)**			
[0-10)	2,444 (58%)	2,280 (57%)	164 (59%)
[10-30)	810 (19%)	751 (19%)	59 (21%)
[30-50)	344 (8.1%)	326 (8.2%)	18 (6.5%)
[50-193]	178 (4.2%)	165 (4.2%)	13 (4.7%)
Unknown	473 (11%)	450 (11%)	23 (8.3%)
**Smoking status**			
Non	2,237 (53%)	2,140 (54%)	97 (35%)
Former	1,231 (29%)	1,130 (28%)	101 (36%)
Current	696 (16%)	621 (16%)	75 (27%)
Unknown	85 (2.0%)	81 (2.0%)	4 (1.4%)
**Permanent makeup**	108 (2.6%)	0 (0%)	108 (45%)

[1] n (%); Median interquartile range (IQR); [] The number is inside the range; () The number is not included.
