# Supplementary material for: Characteristics of a tattooed population and a possible role of tattoos as a risk factor for chronic diseases: Results from the LIFE-Adult-Study
Source: PLoS One. 2025 Sep 9;20(9):e0319229. doi: 10.1371/journal.pone.0319229 (PMC12419626; doi:10.1371/journal.pone.0319229)
Supplement: S3 Table — (PDF) [file pone.0319229.s012.pdf]

S3 Table. Characteristics of tattooed and non-tattooed participants with liver values indicative for a hepatocellular damage.

	Tattooed	Non-tattooed
**Prevalence**	23/203 (11%)	382/3944
**Sex (prevalence among sex)**	Men 16/23 (16/85 men, 19%)<br>Women 7/23 (7/118 women, 6%)	Men 205/382 (205/1946 men, 11%)<br>Women 177/382 (177/1998 Women, 9%)
**T or PMU**	Tattoo 18/23<br>PMU 5/23	-
**Tattooing extent**	Small – 14/23<br>Medium – 3/23<br>Large – 5/23<br>Very large – 1/23	-
**Colours**	Yellow – 4/23<br>**Orange – 2/23**<br>**Red – 6/23**<br>**Blue – 7/23**<br>**Green – 3/23**<br>**Brown – 1/23**<br>**Black – 18/23**	-
**Medical complication rel. to tattoo**	1/23	-
**Median age at baseline (IQR) in years**	48 (45, 60)	59 (50, 65)
**Smoking status** Current<br>Former<br>Non	7 (30%)<br>9 (39%)<br>7 (30%)	53 (14%)<br>139 (36%)<br>188 (49%)<br>2 (0.5%)
**BMI Category**	1 – 0<br>2 – 7 (30%)<br>3 – 11 (48%)<br>4 – 5 (22%)	1 – 1 (0.3%)<br>2 – 44 (12%)<br>3 – 160 (42%)<br>4 – 177 (46%)<br>Unknown – 0
**Alcohol consumption**	6 (1,32) 21.68 g/day	6 (1,26) 14.93 g/day
**Socioeconomic status**	1 – 4 (17%)<br>2 – 17 (74%)<br>3 – 2 (8.7%)	1 – 56 (15%)<br>2 – 239 (63%)<br>3 – 87 (23%)
