# Supplementary material for: Characteristics of a tattooed population and a possible role of tattoos as a risk factor for chronic diseases: Results from the LIFE-Adult-Study
Source: PLoS One. 2025 Sep 9;20(9):e0319229. doi: 10.1371/journal.pone.0319229 (PMC12419626; doi:10.1371/journal.pone.0319229)
Supplement: S4 Table — For the evaluation, only participants from which tattoos were present before answering the questionnaires and cancer diagnosis were included. (PDF) [file pone.0319229.s013.pdf]

S4 Table. Tattooed participants with a diagnosed non-melanoma skin cancer (results from basic and follow-up examinations combined). For the evaluation, only participants from which tattoos were present before answering the questionnaires and cancer diagnosis were included.

**Prevalence**	5/252 (2.0%)	
**T or PMU**	3/5 Tattoo	2/5 PMU
**Tattooing extent**	Tattoo:<br>2/3 medium<br>1/3 large	PMU:<br>2/2 small
**Colours**	Tattoo: 2/3 blue, 1/3 black	PMU: 1/2 black, 1/2 green
**Medical complication rel. to tattoo?**	no 5/5	
**Years between tattoo – cancer, median (IQR)**	19 (47)	
**Median age at diagnosis (IQR) in years**	62 (22)	
**Sex**	Men 2/5<br>Women 3/5	
**Diagnosed cancer free?**	4/5	
**Smoking status**	Current 0/5<br>Former 2/5<br>Non: 3/5	
**BMI Categories**	1 – 0/5<br>2 – 3/5<br>3 – 1/5<br>4 – 1/5	
